# Curcumin in Alzheimer’s Disease: From Mechanistic Insights to Translational Challenges and Emerging Curcuminoid Strategies

**DOI:** 10.3390/ijms27135754

**Published:** 2026-06-25

**Authors:** Katarzyna Stępnik

**Affiliations:** Department of Physical Chemistry, Institute of Chemical Sciences, Faculty of Chemistry, Maria Curie–Skłodowska University in Lublin, Pl. M. Curie-Skłodowskiej 3, 20-031 Lublin, Poland; katarzyna.stepnik@umcs.pl

**Keywords:** curcumin, Alzheimer’s disease, neuroinflammation, oxidative stress, amyloid-β, tau pathology, mitochondrial dysfunction, bioavailability, formulation strategies, bisdemethoxycurcumin, curcuminoids, translational research

## Abstract

Alzheimer’s disease (AD) is a multifactorial neurodegenerative disorder driven by complex interactions between protein aggregation, oxidative stress, neuroinflammation, and cellular dysfunction. Among plant-derived compounds, curcumin has emerged as one of the most extensively studied polyphenols due to its broad spectrum of biological activities. This review provides a critical synthesis of the mechanistic, preclinical, and clinical evidence on curcumin in AD. Experimental studies consistently demonstrate that curcumin modulates key pathogenic processes, including neuroinflammatory signaling, oxidative stress, and amyloid-β aggregation, with more limited evidence for effects on tau pathology. While in vitro studies offer detailed mechanistic insights, in vivo models provide more integrated evidence, including improvements in cognitive performance and reductions in pathological markers. Despite this strong preclinical foundation, the clinical evidence remains limited and inconsistent. Randomized controlled trials have not demonstrated clear therapeutic efficacy, with outcomes strongly influenced by formulation, bioavailability, and study design. Poor solubility, rapid metabolism, and limited brain exposure remain key translational barriers. In response, increasing attention has been directed toward formulation strategies and structurally related compounds. Emerging curcuminoids, such as bisdemethoxycurcumin (BDMC), are discussed as potential next-generation candidates. Preliminary evidence suggests that BDMC may modulate oxidative stress, autophagy, astrocyte senescence, and amyloid-related processes, although the data remain largely preclinical. Overall, curcumin represents a mechanistically rich and preclinically promising multi-target compound but with unresolved translational limitations. Future research should prioritize pharmacokinetic optimization, formulation-dependent validation, and exploration of novel curcuminoid strategies to bridge the gap between experimental findings and clinical application in AD.

## 1. Introduction

Alzheimer’s disease is the most prevalent neurodegenerative disorder with a multifactorial etiology and one of the main causes of dementia worldwide. At the neuropathological level, AD is characterized by the presence of extracellular amyloid-β (Aβ)-containing plaques and intracellular neurofibrillary tangles (NFTs) composed of abnormal hyperphosphorylated tau, but its clinical and pathological expression involves a much more complex disease process than the classical amyloid–tau framework [1,2]. More recent models propose AD as a multifactorial disorder with interacting disturbances in proteostasis, lysosomal and endosomal trafficking, synaptic homeostasis, mitochondrial function, oxidative balance, and innate immune signaling [3,4], and therapeutic efforts have increasingly shifted from single-target concepts to strategies able to modulate multiple pathogenic processes simultaneously [3,5]. Among the numerous interacting mechanisms implicated in AD, oxidative stress and neuroinflammation have emerged as central and interdependent drivers of disease progression [6,7,8]. Oxidative stress is characterized by an imbalance between reactive oxygen species (ROS) production and antioxidant defenses, resulting in lipid, protein, and nucleic acid damage and contributing to synaptic dysfunction and neuronal loss [9,10]. Simultaneously, inflammatory responses mediated by brain-resident glial cells are now recognized as central to AD pathogenesis rather than just secondary reactions to neuronal injury [11,12]. Importantly, oxidative stress and neuroinflammation reinforce each other, creating a self-perpetuating pathogenic loop that may accelerate neurodegeneration [13,14,15].

This conceptual shift toward a multifactorial and network-based understanding of AD is particularly relevant for the evaluation of plant polyphenols as potential neuroprotective agents. Rather than acting only as direct radical scavengers, polyphenols are widely discussed as pleiotropic modulators of interconnected cellular networks, influencing signaling pathways, neuroinflammation, mitochondrial function, and the brain–gut axis [16,17]. More recent reviews also emphasize that their relevance to brain health cannot be reduced to antioxidant activity alone and that their biological effects may involve transcriptional, metabolic, and immunomodulatory mechanisms [17,18,19]. This broader framework supports the rationale for focusing on selected polyphenols as multi-target therapeutic candidates in Alzheimer’s disease.

Among these compounds, curcumin has attracted particular attention because it has been reported to modulate several pathological mechanisms implicated in AD, including oxidative stress, neuroinflammation, amyloid-β aggregation, tau pathology, mitochondrial dysfunction, and dysregulated cellular signaling pathways [20,21,22]. This broad mechanistic profile provides a strong rationale for investigating curcumin as a potential multi-target neuroprotective agent in Alzheimer’s disease.

## 2. Neuroinflammation in Alzheimer’s Disease

It is now commonly acknowledged that neuroinflammation plays a key role in the pathophysiology of Alzheimer’s disease, contributing to both the onset and progression of the illness. Inflammatory processes are increasingly seen as active drivers of disease dynamics, closely interacting with Aβ accumulation, tau pathology, and synaptic dysfunction, rather than as a secondary response to neuronal degeneration [7,13]. Genetic, transcriptomic, and experimental data demonstrating the crucial role of innate immune mechanisms in AD have provided compelling evidence for this paradigm shift [23,24]. As the innate immune system was shown to play a causal role in neurodegeneration, this paradigm shift sparked a surge in interest in microglia biology. Meanwhile, microglial activation, which had already been shown in the initial AD neuropathology studies, was thought to be a component of an inflammatory response that followed neuron damage [24].

Microglia, the resident immune cells of the central nervous system, play a central role in coordinating neuroinflammatory responses. Under physiological conditions, they contribute to the maintenance of brain homeostasis through continuous surveillance, phagocytosis, and the secretion of trophic factors. In the early stages of Alzheimer’s disease, microglial activation may exert protective effects by promoting amyloid-β clearance and limiting plaque formation [13,25].

However, prolonged exposure to pathological stimuli drives microglia toward dysfunctional activation states characterized by reduced phagocytic capacity and increased production of pro-inflammatory mediators, including tumor necrosis factor-α (TNF-α), interleukin-1β (IL-1β), and interleukin-6 (IL-6) [7,26]. These changes are associated with neuronal damage and synaptic dysfunction.

The traditional binary classification of microglia into M1 (pro-inflammatory) and M2 (anti-inflammatory) phenotypes has been increasingly challenged, with emerging evidence supporting a continuum of activation states [27]. In this context, the concept of disease-associated microglia (DAM) has emerged as a more accurate representation of microglial responses in neurodegeneration. Single-cell transcriptomic studies have demonstrated that DAM exhibit distinct transcriptional profiles and are closely associated with amyloid plaques. Importantly, both protective and detrimental roles have been described as depending on disease stage and microenvironmental context, which highlights the dynamic and context-dependent nature of microglial function [28].

Additional genetic evidence further supports the role of microglia in Alzheimer’s disease. Variants of the triggering receptor expressed on the myeloid cells 2 (*TREM2*) gene have been identified as significant risk factors for late-onset AD. TREM2 plays a key role in regulating microglial metabolism, survival, and phagocytic activity, particularly in response to lipid and amyloid-related signals [29,30]. Impaired TREM2 signaling has been associated with defective plaque compaction and altered microglial responses, which underscores its importance in the immune pathways linked to disease progression [31,32].

Astrocytes, together with microglia, are central components of the neuroinflammatory environment in AD. Reactive astrocytes display considerable heterogeneity and may adopt both protective and neurotoxic phenotypes. Notably, activated microglia can induce a neurotoxic astrocytic state through the release of inflammatory mediators, leading to neuronal dysfunction and loss of synaptic support [33,34,35]. Bidirectional communication between microglia and astrocytes represents a key mechanism that amplifies inflammatory signaling and sustains chronic neuroinflammation [13,33,36].

At the molecular level, neuroinflammatory responses in AD are regulated by multiple signaling pathways. Among these, the nuclear factor kappa B (NF-κB) pathway plays a central role in promoting pro-inflammatory gene expression [7]. In parallel, amyloid-β accumulation has been linked to activation of the NLRP3 inflammasome, which results in the maturation of IL-1β and IL-18. Experimental studies indicate that NLRP3 activation contributes to AD pathology, whereas its inhibition may attenuate neuroinflammation and improve disease outcomes in animal models [37,38,39,40].

Importantly, neuroinflammation is closely interconnected with other pathogenic mechanisms, particularly oxidative stress. Inflammatory mediators exacerbate oxidative damage and mitochondrial dysfunction, while reactive oxygen species can further activate inflammatory pathways such as NF-κB and inflammasome signaling. This mutually reinforcing cycle accelerates neuronal injury and disease progression [6,7,23].

Neuroinflammation also contributes to the development of core Alzheimer’s disease pathologies. Inflammatory mediators have been shown to influence the processing of amyloid precursor protein, promote amyloid-β aggregation, and facilitate tau hyperphosphorylation and propagation [7,13]. Accordingly, immune dysregulation not only represents a response to ongoing pathology but also actively contributes to its progression [41].

Neuroinflammation can, therefore, be viewed as a multifactorial process involving coordinated interactions between intracellular signaling pathways, glial cells, and genetic risk factors. This complexity supports the rationale for therapeutic strategies targeting multiple components of the inflammatory cascade [42].

In this context, plant-derived polyphenols have attracted considerable attention due to their ability to modulate redox balance, regulate key inflammatory pathways, and influence cell–cell communication within the neuroinflammatory network [18,43,44,45].

## 3. Oxidative Stress in Alzheimer’s Disease

Oxidative stress is widely recognized as a major contributor to the pathogenesis of Alzheimer’s disease and plays a significant role in neuronal dysfunction and degeneration [9,10]. It arises from an imbalance between the production of reactive oxygen species and the capacity of cellular antioxidant defense systems, leading to damage to lipids, proteins, and nucleic acids. The brain is particularly vulnerable to oxidative stress due to its high oxygen consumption and lipid-rich composition, which makes redox imbalance a critical factor in the disruption of neuronal homeostasis and synaptic function [6].

Multiple sources contribute to ROS overproduction in AD, among which mitochondrial dysfunction is one of the most prominent. Impairments in mitochondrial respiratory chain activity compromise neuronal energy metabolism, resulting in increased electron leakage, enhanced ROS generation, and reduced ATP production [46,47]. Notably, mitochondrial abnormalities have been observed early in the course of AD, which suggests that oxidative stress may actively contribute to disease onset rather than merely representing a consequence of neurodegeneration [48,49].

In addition to mitochondrial sources, amyloid-β can directly induce oxidative stress. Aβ aggregation has been shown to enhance oxidative damage through free radical generation and interactions with redox-active metal ions such as copper and iron [50]. This oxidative environment promotes lipid peroxidation and protein oxidation, ultimately contributing to neuronal loss and synaptic dysfunction. Moreover, oxidative stress can further enhance Aβ production and aggregation, creating a self-amplifying pathogenic loop [6,50].

Oxidative damage is also closely associated with tau pathology. Elevated ROS levels can promote tau hyperphosphorylation through the activation of stress-related kinases, leading to microtubule destabilization and neurofibrillary tangle formation [51,52]. These findings indicate that oxidative stress not only contributes to neuronal injury but also actively modulates core pathological features of AD [53,54].

At the cellular level, antioxidant defense systems play a critical role in maintaining redox homeostasis. Among these, the nuclear factor erythroid 2-related factor 2 (Nrf2) pathway is a key regulator of antioxidant gene expression. Under physiological conditions, Nrf2 activation promotes the transcription of genes involved in redox balance and detoxification. However, accumulating evidence suggests that Nrf2 signaling is impaired in AD, which increases susceptibility to oxidative damage. Consequently, restoration of Nrf2 activity has been proposed as a potential therapeutic strategy [55,56].

Oxidative stress is closely interconnected with neuroinflammation. Reactive oxygen species can activate inflammatory signaling pathways, including NF-κB, while inflammatory mediators further enhance ROS production, forming a self-perpetuating cycle of damage. This interaction highlights the need to consider oxidative stress and neuroinflammation as integrated components of AD pathophysiology rather than independent processes [6,13].

Overall, oxidative stress represents a complex and dynamic contributor to AD, affecting cellular signaling pathways, protein aggregation, and mitochondrial function. This complexity underscores the need for therapeutic approaches capable of targeting multiple aspects of redox imbalance. In this context, plant-derived polyphenols are of particular interest due to their ability to modulate redox-sensitive signaling pathways, support mitochondrial function, and act as antioxidants [16,19,43,57].

## 4. Curcumin as a Modulator of Neuroinflammation and Oxidative Stress in Alzheimer’s Disease

### 4.1. General Rationale for Curcumin in AD

Curcumin, the principal polyphenolic component of *Curcuma longa*, remains one of the most extensively studied phytochemicals in the context of Alzheimer’s disease. Its reported biological activities include modulation of neuroinflammation, oxidative stress, amyloid-β aggregation, tau-related pathology, mitochondrial dysfunction, and cell-survival signaling pathways. Recent reviews consistently characterize curcumin as a multi-target compound rather than a simple antioxidant [20,21,22,58,59,60]. The complexity and interdependence of the pathogenic processes in AD make this multi-target profile especially pertinent. Curcumin’s neuroprotective potential is supported by a wealth of preclinical data, but its clinical translation is still limited. This is primarily because of its poor oral bioavailability, quick metabolism, and restricted brain exposure, all of which continue to be significant obstacles to clinical application [61,62,63,64].

### 4.2. Anti-Inflammatory Effects

Attenuation of inflammatory signaling is a consistently reported feature of curcumin activity. In Alzheimer’s disease, chronic activation of astrocytes and microglia is increasingly recognized as an active driver of disease progression rather than merely a response to neuronal damage [7,13,65].

In this context, curcumin has been widely reported to modulate neuroinflammatory pathways, particularly those involving NF-κB signaling, cytokine production, oxidative stress–inflammation crosstalk, and glial activation [66,67,68].

At the molecular level, its anti-inflammatory effects are most commonly linked to suppression of NF-κB-associated signaling. NF-κB is a key transcriptional regulator of pro-inflammatory gene expression, driving the production of mediators such as tumor necrosis factor-α (TNF-α), interleukin-1β (IL-1β), interleukin-6 (IL-6), inducible nitric oxide synthase (iNOS), and cyclooxygenase-2 (COX-2). Reviews consistently report that curcumin can attenuate NF-κB-dependent inflammatory responses, although the strength of this evidence remains largely preclinical [68,69].

This mechanism is supported by direct in vitro evidence in microglia-like systems. In BV-2 mouse microglial cells stimulated with lipoteichoic acid (LTA), curcumin reduced the expression of iNOS and cyclooxygenase-2 (COX-2) and inhibited the release of inflammatory mediators, including tumor necrosis factor-alpha (TNF-α), prostaglandin E2, and nitric oxide. These effects were associated with activation of the antioxidative HO-1/Nrf2/ARE pathway and inhibition of p38 mitogen-activated protein kinase (MAPK) signaling, which suggests coordinated regulation of inflammatory and redox-sensitive pathways rather than a single-target mechanism [68,70].

Additional in vitro studies indicate that curcumin can modulate microglial inflammatory responses through alternative signaling pathways. For example, modulation of the Janus kinase/signal transducer and activator of transcription/suppressor of cytokine signaling (JAK/STAT/SOCS) axis has been reported in BV-2 cells, which further supports a multi-target mode of action [71]. However, given the variability in experimental models and conditions, these findings should be interpreted with caution.

Stronger integrative evidence comes from in vivo models. A study in an Aβ1–42-induced Alzheimer’s disease mouse model reported that curcumin improved behavioral outcomes and reduced inflammatory and oxidative stress markers, including TNF-α, IL-6, IL-1β, superoxide dismutase, and malondialdehyde, and was associated with activation of the AMPK signaling pathway [72]. These findings are particularly relevant as they integrate inflammatory, oxidative, and metabolic pathways within a single experimental context.

Additional animal studies support the anti-neuroinflammatory profile of curcumin. In a p25 transgenic mouse model, curcumin attenuated glial activation, reduced the production of pro-inflammatory mediators, and improved cognitive outcomes, although such models do not fully recapitulate the complexity of human Alzheimer’s disease [73].

Taken together, these findings indicate that curcumin acts as a preclinically active modulator of neuroinflammatory signaling. Mechanistic support is strongest in in vitro systems, whereas integrated effects are more consistently demonstrated in animal models. However, direct clinical evidence for anti-neuroinflammatory efficacy in AD remains limited, and translation to human disease has yet to be established.

### 4.3. Impact on Redox Homeostasis and Oxidative Stress

Oxidative stress represents one of the most consistently reported mechanistic features of curcumin in dementia research. A recent systematic review focusing on preclinical models reported that curcumin treatment was consistently associated with reductions in malondialdehyde levels, alongside improvements in antioxidant defenses and cognitive outcomes [58]. Although this analysis was not restricted to Alzheimer’s disease, it provides strong support for the broader conclusion that modulation of oxidative stress is a robust and reproducible preclinical effect of curcumin.

Importantly, curcumin’s antioxidant activity is not limited to direct radical scavenging but also involves modulation of redox-sensitive signaling pathways, including Nrf2-mediated responses [74]. This broader mechanistic profile aligns with the current understanding of polyphenols as regulators of cellular signaling rather than simple chemical antioxidants.

Evidence from meta-analyses further indicates that curcumin reduces oxidative stress markers such as malondialdehyde while enhancing endogenous antioxidant defenses, including superoxide dismutase and glutathione-related systems [75].

Preclinical evidence also highlights a strong interplay between oxidative stress and inflammation in curcumin-treated models. Reductions in oxidative stress markers are frequently accompanied by decreased inflammatory activity and improved cognitive performance in rodent AD-like systems. These findings are consistent with the broader framework of AD pathophysiology, in which reactive oxygen species and neuroinflammatory processes form a self-amplifying pathogenic loop.

### 4.4. Effects on Amyloid-β Pathology

The anti-amyloid activity of curcumin represents one of the most extensively studied aspects of its biological profile. Experimental studies consistently demonstrate that curcumin can directly interact with amyloid-β, inhibit fibril formation, and destabilize pre-formed aggregates [76,77]. In addition to these direct interactions, curcumin has been shown to influence aggregation dynamics and reduce amyloid burden in vivo, including decreased plaque deposition in transgenic mouse models [78,79].

However, more recent literature adopts a more cautious perspective compared to earlier reports. Rather than describing a uniform inhibition of Aβ fibrilization, current evidence suggests that curcumin exerts context- and model-dependent effects on aggregation pathways, assembly states, and aggregate toxicity [80]. This shift reflects a broader recognition that amyloid modulation is a complex, dynamic process rather than a single-target mechanism.

Evidence from both in vitro and in vivo studies supports this interpretation. Curcumin has long been shown to bind amyloidogenic molecules [81], and ongoing research continues to explore derivatives and analogs designed to improve potency and activity at lower concentrations. In animal models, particularly those employing bioavailable formulations, curcumin has been associated with improvements in amyloid-related pathological readouts and behavioral outcomes, although these effects are often accompanied by parallel changes in inflammatory and oxidative stress markers [82,83,84].

Despite robust preclinical findings, translation to human disease remains limited. Clinical studies in patients with Alzheimer’s disease have not demonstrated clear anti-amyloid efficacy, and outcomes have been largely constrained by pharmacokinetic limitations and study design factors [85]. The strongest human imaging study reported changes in PET-derived measures related to amyloid and tau in non-demented individuals receiving a bioavailable curcumin formulation; however, these findings do not constitute evidence of disease-modifying efficacy in AD [86].

Taken together, the available evidence supports a well-established anti-amyloid profile of curcumin in experimental systems while highlighting the absence of convincing clinical confirmation. This discrepancy underscores the importance of distinguishing between mechanistic potential and translational efficacy when evaluating curcumin as a candidate therapeutic agent in Alzheimer’s disease [85,87].

### 4.5. Effects on Tau Pathology

Curcumin’s effects on tau have received increasing attention in recent years, in part because research on Alzheimer’s disease has moved beyond a framework that is solely focused on amyloid. Curcumin should be considered in relation to tau hyperphosphorylation, tau aggregation, and tau-associated neurotoxicity in addition to its effects on amyloid-β, according to a specialized review on curcumin and tau pathology [88]. This viewpoint is pertinent because tau pathology is strongly associated with neurodegeneration and cognitive decline in AD, which makes tau-directed mechanisms a crucial part of any discussion about multi-target therapy. In vitro aggregation studies provide the most compelling direct evidence of curcumin’s tau-directed effects. It has been demonstrated that curcumin binds tau and prevents tau fibrilization and aggregation in vitro. Curcumin was found to have a direct impact on tau assembly and aggregate stability by inhibiting the formation of β-sheets, reducing the formation of tau fibrils, and disintegrating preformed tau filaments [89]. Although these results should be viewed as biochemical evidence rather than proof of therapeutic efficacy in vivo, they offer significant mechanistic support for the notion that curcumin can disrupt tau aggregation pathways. The potential of curcumin to modulate tau is further supported by more recent formulation-based and derivative studies. The anti-tau activity of curcumin or curcumin-containing systems may be enhanced by formulation strategies, as a curcumin–artemisinin co-amorphous system was found to inhibit recombinant tau aggregation in vitro and modulate tau phosphorylation-related readouts [90]. Similarly, in cellular models such as human-derived neuroblastoma (SH-SY5Y) cells and primary cortical neurons, newly synthesized curcumin derivatives have been demonstrated to modify toxic tau oligomer aggregation pathways and transform toxic tau oligomers into less toxic aggregate species [91]. Importantly, the curcumin derivatives investigated in these studies were specifically designed to overcome some of the physicochemical limitations of native curcumin. For example, Lo Cascio et al. [91] evaluated several synthetic curcumin analogs, including the curcumin-like derivatives CL3 and CL8, the oxadiazole-containing derivative CH8, the hemi-curcuminoid HemiC9, and the Calebin-A analogs Cal7 and Cal9. All of these compounds were characterized by removal of the β-diketone moiety, a structural feature considered to contribute to the poor aqueous solubility and limited bioavailability of native curcumin. The derivatives were shown to remodel toxic tau oligomers into larger, less toxic aggregate species in cellular models [91].

Curcumin may also affect tau hyperphosphorylation, according to evidence from animal models, though this evidence is more sparse and inconsistent than that found in the literature on oxidative stress and neuroinflammation. Curcumin decreased tau hyperphosphorylation and downregulated glycogen synthase kinase 3 (GSK3) and cyclin-dependent kinase 5 (CDK5), linked to aberrant tau phosphorylation, in a scopolamine-induced AD-like rat model [92]. Curcumin treatment decreased p25-mediated tau hyperphosphorylation in a p25 transgenic mouse model, according to a review of tau-focused curcumin studies [73]. This suggests that curcumin may indirectly influence tau pathology through kinase-related and stress-response pathways [88].

Despite these results, claims about tau should be presented more carefully than claims about the anti-inflammatory or antioxidant properties of curcumin. A large portion of the direct tau evidence is derived from cellular models, simplified aggregation assays, or purified protein systems, which are useful for identifying mechanisms but do not accurately replicate the intricacy of tau pathology in the human brain. Furthermore, it is challenging to identify tau-specific mechanisms because in vivo studies frequently evaluate tau-related endpoints alongside Aβ, oxidative stress, inflammation, or behavioral outcomes. When considered collectively, the evidence suggests that curcumin has the ability to modulate tau in preclinical systems, especially by inhibiting tau aggregation, remodeling toxic tau assemblies, and potentially controlling tau phosphorylation pathways [89]. Nevertheless, the majority of this evidence is still preclinical and mechanistic. There is currently no solid clinical proof that curcumin alters tau pathology in AD patients. Therefore, rather than being a clinically proven anti-tau treatment, curcumin should be characterized as a promising tau-modulating substance in experimental models [88,89].

### 4.6. Mitochondrial Function, Apoptosis, and Cellular Resilience

The concept of cellular resilience, which encompasses mitochondrial function, apoptosis regulation, oxidative stress responses, and neuronal survival pathways, is increasingly being used to discuss curcumin. This viewpoint is especially pertinent to Alzheimer’s disease, where the production of reactive oxygen species, decreased bioenergetics, synaptic failure, and activation of apoptotic pathways are all strongly associated with mitochondrial dysfunction. In this regard, curcumin is increasingly regarded as both an antioxidant and a modulator of downstream stress-response mechanisms that affect the survival and vulnerability of neurons [93]. Curcumin may offer protection against mitochondrial dysfunction linked to AD-like pathology, according to experimental research. For instance, attenuation of mitochondrial dysfunction and apoptosis has been associated with curcumin-mediated neuroprotection against Aβ-induced toxicity, which supports the idea that mitochondrial stabilization may contribute to its neuroprotective profile [94].

Furthermore, a more recent study found that curcumin slowed the progression of AD-like conditions by modifying mitochondrial stress responses via the axis of Jumonji domain-containing protein 3 (JMJD3)-trimethylated lysine 27 on histone H3 (H3K27me3)-brain-derived neurotrophic factor (JMJD3–H3K27me3–BDNF) [95]. This suggests that curcumin’s effects might go beyond direct redox activity to include regulation of epigenetic and mitochondrial stress responses. Additionally significant is the connection between apoptosis and mitochondrial defense. Mitochondrial damage can increase cytochrome c release, caspase activation, and neuronal death in AD-related models. In an Aβ1–42-induced AD mouse model, curcumin has been shown to improve cognition, decrease inflammatory cytokine levels, decrease malondialdehyde, and increase superoxide dismutase activity in addition to reducing apoptosis-associated brain tissue damage [72]. Because they link oxidative and inflammatory readouts with mitochondrial/apoptotic injury within the same experimental framework, these findings are especially pertinent.

Curcumin may play a part in controlling damage caused by endoplasmic reticulum stress and mitochondria, according to cell-based research. Curcumin has been shown to prevent thapsigargin-induced cell damage and apoptosis in SH-SY5Y cells, with mitofusin-2-related mitochondrial dysfunction involved [96]. Because endoplasmic reticulum stress, mitochondrial dysfunction, and apoptosis are interrelated processes linked to neuronal injury, this model is mechanistically relevant even though it is not specifically an AD model. Therefore, rather than providing concrete proof of disease modification in AD, such studies should be interpreted as mechanistic support. According to the available data, curcumin may promote cellular resilience by reducing apoptosis-related damage, regulating mitochondrial stress, and affecting signaling pathways linked to survival. Nevertheless, these results are frequently assessed in conjunction with alterations in oxidative stress, inflammation, Aβ burden, or behavioral performance. Therefore, rather than being separate therapeutic mechanisms, curcumin’s mitochondrial and anti-apoptotic effects should be understood as a component of an interconnected neuroprotective network [87,97,98].

### 4.7. In Vitro Studies

In vitro studies provide the foundation for mechanistic understanding of curcumin activity; however, their translational relevance is inherently limited. The literature on curcumin in AD is mechanistically rich and has been crucial in identifying its possible molecular targets. However, the available evidence is methodologically heterogeneous. Curcumin’s effects on Aβ aggregation, tau protein aggregation, oxidative stress, inflammatory mediator release, mitochondrial dysfunction, and neuronal viability have been studied in both cellular and cell-free systems. These investigations are important because they enable the controlled investigation of mechanistic questions, such as whether curcumin can directly interact with amyloidogenic proteins, modify redox-sensitive pathways, or affect glial inflammatory signaling. Some of the most convincing mechanistic evidence comes from in vitro research on Aβ. Curcumin has been demonstrated to directly interact with amyloidogenic species by inhibiting Aβ aggregation and destabilizing preformed fibrils in biochemical systems [79,83,99].

The entire cellular and extracellular environment of the human AD brain cannot be replicated by these assays, which usually use simplified protein systems. Redox-modulating and anti-inflammatory mechanisms are also supported by in vitro research. Curcumin decreased the release of inflammatory mediators and altered p38 MAPK signaling and HO-1/Nrf2/ARE pathways in BV-2 microglial cells [70]. The idea that curcumin can affect inflammatory signaling through a variety of pathways is further supported by additional research in BV-2 cells that revealed modulation of the Janus kinase/signal transducer and activator of transcription/suppressor of cytokine signaling (JAK/STAT/SOCS) axis [71].

Although these findings support curcumin’s mechanistic plausibility as an anti-neuroinflammatory substance, they still depend on the type of cell, stimulus, concentration, and duration of exposure. Tau-directed effects have also been better understood thanks to in vitro research. Research employing cellular models or purified tau indicates that curcumin and its derivatives may disrupt tau aggregation or alter harmful tau oligomeric species [68,89,91]. These results should not be taken as proof of clinical anti-tau efficacy, but they are mechanistically instructive, particularly for comprehending how curcumin-like compounds may impact protein assembly. The fact that curcumin concentrations used in experiments frequently surpass levels attainable in human plasma or brain tissue following traditional oral administration is a major drawback of in vitro research. Given curcumin’s low systemic exposure, quick metabolism, and poor aqueous solubility, this problem is particularly crucial. Because of this, in vitro results should not be viewed as independent proof of therapeutic relevance but rather as evidence of biological plausibility and mechanism discovery. As a result, validation in animal models and formulation techniques intended to enhance pharmacokinetic performance are becoming more and more important in contemporary curcumin research.

### 4.8. In Vivo Studies

In vivo models offer greater translational relevance than in vitro systems because they integrate pharmacokinetics, tissue exposure, behavioral outcomes, neuropathological changes, oxidative stress, inflammation, and neuronal injury within a living organism. Although preclinical and inconsistent, the in vivo research on curcumin in AD and dementia models is generally positive. The idea that curcumin has repeatable positive effects in rodent dementia models is supported by recent systematic research [73,78]. In addition to summarizing improvements in antioxidant defenses and cognitive outcomes, a systematic review of 29 rodent studies found that curcumin consistently reduced oxidative stress markers, with all studies evaluating malondialdehyde reporting significant reductions. These findings identified oxidative stress modulation as one of the most reliable preclinical effects of curcumin, although the review was not limited exclusively to AD [58]. This profile is further supported by in vivo studies that are specific to AD. Curcumin enhanced cognitive and spatial memory outcomes, decreased oxidative stress markers and inflammatory cytokines, attenuated pathological damage and apoptosis in brain tissue, and was linked to AMPK pathway activation in an Aβ1–42-induced AD mouse model [37,73,78,82].

Additional animal studies also emphasize that improved formulation does not automatically translate into beneficial neuropathological outcomes. In a study using a bioavailable micellar curcumin formulation administered in drinking water to AD transgenic mice, the treatment did not alter amyloid plaque size or number and did not modify mechanisms regulating amyloid-β production. Although the formulation was designed to improve oral bioavailability, the study reported increased total brain levels of glial fibrillary acidic protein (GFAP), suggesting a possible increase in neuroinflammatory activity [100]. This finding highlights an important translational caveat: enhanced systemic delivery may increase biological exposure without necessarily producing disease-modifying effects in the brain. Moreover, the observed increase in GFAP highlights the possibility that enhanced systemic exposure achieved through formulation strategies may not uniformly translate into anti-inflammatory effects and may generate context-dependent glial responses in models with established pathology. Therefore, micellar and other delivery-enhanced curcumin systems should be evaluated not only for pharmacokinetic improvement and amyloid-related outcomes but also for neuroinflammatory safety endpoints, including astrocytic and microglial activation [100].

Because they demonstrate that curcumin’s effects are rarely limited to a single pathway, in vivo studies are also important. Improvements in oxidative stress, inflammatory markers, Aβ burden, tau-related endpoints, apoptosis, or mitochondrial function frequently coexist with improvements in cognition in animal models. Curcumin’s multi-target interpretation is supported by this pattern, but it also poses a methodological challenge: it can be challenging to identify whether redox modulation, anti-inflammatory effects, anti-amyloid activity, mitochondrial protection, or a combination of these mechanisms is the main driver of cognitive improvement.

The animal-related literature is still quite varied. Aβ peptide injection paradigms, transgenic mice, chemically induced dementia-like models, diet-related models, and models of aging-related cognitive impairment are among the models. Importantly, several factors may contribute to the inconsistent outcomes reported across animal studies. First, curcumin exhibits limited blood–brain barrier penetration and rapid systemic metabolism, meaning that improvements in oral bioavailability do not necessarily result in therapeutically relevant concentrations within the brain [64,101,102]. Second, different AD animal models reproduce distinct aspects of the disease, including amyloid pathology, tau pathology, neuroinflammation, metabolic dysfunction, and age-related cognitive decline, which may differentially influence responsiveness to curcumin treatment [73,78,94,101]. Third, the stage of disease at treatment initiation may substantially affect outcomes, as interventions administered during the preclinical or early stages of pathology may yield different responses than those introduced after extensive neuropathological changes have become established [85]. Additional variability arises from differences in dosage regimens, treatment duration, routes of administration, behavioral testing protocols, curcumin formulations, and outcome measures, factors that have been recognized as important contributors to heterogeneity across preclinical studies and meta-analyses [58,103]. Collectively, these factors likely explain a substantial proportion of the variability and inconsistencies reported across in vivo studies of curcumin in AD-related models.

Therefore, although the overall direction of the evidence from animal studies is generally favorable, the heterogeneity of the experimental models and study designs precludes strong clinical conclusions without appropriate qualification. Nevertheless, the available in vivo evidence consistently supports the view that curcumin exerts multi-target activity in AD-like pathology, with the strongest effects observed for oxidative stress, neuroinflammation, and cognitive outcomes [87,104]. Additional evidence also suggests beneficial effects on apoptosis, mitochondrial dysfunction, and proteinopathy-related endpoints. However, these findings remain preclinical, and their translational relevance depends substantially on the extent to which individual models replicate human AD pathology, as well as on differences in formulation, dose, and tissue exposure. Consequently, despite generally encouraging results, direct extrapolation to human disease should be approached with caution [104].

### 4.9. Clinical Studies and Translational Relevance

Compared to the preclinical literature, the clinical evidence for curcumin in Alzheimer’s disease is still scarce and significantly weaker. A 24-week randomized, double-blind, placebo-controlled study in patients with mild-to-moderate AD, followed by an open-label extension, is the most well-known dedicated clinical trial in diagnosed AD. Although oral curcumin C3 complex was generally well tolerated in that study, there was no discernible clinical or biomarker efficacy during the placebo-controlled period [105]. Because it tested curcumin directly in an AD population, this trial is still significant. However, it also highlights the discrepancy between high experimental activity and low efficacy following traditional oral administration.

An 18-month randomized, double-blind, placebo-controlled trial of bioavailable Theracurmin in middle-aged and older non-demented adults provides a different type of clinical evidence. That study examined brain signals related to tau and amyloid using 2-(1-{6-[(2-[F-18]fluoroethyl)(methyl)amino]-2-naphthyl}ethylidene)malononitrile positron emission tomography (FDDNP-PET) and reported improvements in memory and attention. The authors came to the conclusion that daily oral Theracurmin may enhance memory function and that PET results were consistent with decreased tau and amyloid buildup in specific brain regions [84]. However, because the participants in that study were not patients with established AD, it should be interpreted with caution when considering AD therapy. As a result, it offers a biologically intriguing translational signal but no proof that curcumin can effectively treat AD.

The possibility that bioavailable formulations could result in quantifiable cognitive benefits has been investigated in more recent clinical research. CurQfen, a curcumin–galactomannan complex, was found to improve a number of cognitive and functional outcomes when compared to placebo and unformulated standard curcumin in a randomized, double-blind, placebo-controlled study of people with moderate dementia caused by AD [106]. Because it directly addresses formulation-dependent efficacy, that study is pertinent; however, it should be carefully considered in light of the limited clinical evidence base and the requirement for independent replication. A cautious interpretation is also supported by systematic evidence. Both in vivo AD animal models and randomized controlled trials involving human subjects were included in a systematic review and meta-analysis on cognitive aging. Curcumin was found to consistently improve cognition in preclinical animal studies, but the evidence for a strong clinical benefit was not as strong in pooled human data. This supports the more general conclusion that there is a glaring disparity between promising preclinical results and less certain clinical efficacy in the curcumin field [20]. All things considered, recent research on humans indicates that curcumin is generally safe and, depending on the formulation, may result in signals related to cognition or biomarkers. Nevertheless, there is still insufficient, inconsistent, and limited clinical evidence to support curcumin as a successful treatment for AD. Inconsistent results are probably caused by variations in formulation, dose, duration, participant population, disease stage, and outcome measures. Curcumin is, therefore, best characterized as a formulation-sensitive, mechanistically promising candidate whose clinical relevance in AD is still being studied.

### 4.10. Bioavailability and Formulation Challenges

One of the main translational obstacles in curcumin research is still poor bioavailability. After oral administration, native curcumin usually results in low systemic exposure, extensive metabolism, and low aqueous solubility [107]. These restrictions are especially significant for AD, as therapeutic relevance would necessitate adequate exposure to biologically active curcumin species in the central nervous system in addition to gastrointestinal absorption and systemic circulation. The distinction between total curcuminoid exposure, conjugated metabolites, and pharmacologically significant unconjugated curcumin is becoming more and more important in contemporary curcumin research. Following oral administration, curcumin undergoes extensive phase II metabolism in the intestinal wall and liver, predominantly through glucuronidation and sulfation, which results in the formation of curcumin glucuronides and sulfates. These conjugated metabolites generally exhibit lower membrane permeability than free curcumin and are considered less likely to cross the blood–brain barrier efficiently. Consequently, plasma concentrations of pharmacologically relevant unconjugated curcumin remain extremely low following administration of conventional curcumin preparations [61,107].

According to an independent crossover pharmacokinetic study comparing a number of commercial formulations, plasma concentrations of unconjugated curcumin remained extremely low even when formulations increased uptake and produced higher levels of conjugated curcumin. The authors contended that rather than poorly membrane-permeable conjugated metabolites, bioavailability claims should concentrate on biologically significant unconjugated curcumin [108,109,110]. This result implies that greater bioavailability does not always imply therapeutically significant brain exposure and supports a more circumspect interpretation of formulation studies. However, because traditional curcumin exposure is pharmacokinetically unfavorable, formulation strategies are still very important. Numerous strategies intended to enhance absorption are described in reviews of clinical curcumin formulations, such as micelles, phospholipid complexes, lipid-based carriers, nanoparticles, amorphous dispersions, and curcumin–fiber or curcumin–polysaccharide complexes [102,109,110,111,112,113,114,115,116,117,118,119,120,121,122].

Several generations of delivery systems have been developed to improve bioavailability and clinical performance, according to a review of clinical trials using curcumin formulations. It also noted that formulation claims need to be carefully evaluated rather than assuming that all enhanced formulations are equivalent [63]. Because the blood–brain barrier (BBB) separates the central nervous system from systemic circulation, formulation science is particularly crucial for AD. Improved formulation may make it more feasible to target AD-relevant pathology, according to preclinical research employing delivery-enhanced curcumin preparations. Several delivery systems, including micellar formulations, lipid-based nanoparticles, phospholipid complexes, and polysaccharide-based carriers, have demonstrated improved systemic exposure and enhanced brain accumulation of curcumin in animal models compared to native curcumin preparations [63,102,109,111,112,113,115,116,117,118,119,120]. However, in many cases, the distinction between unconjugated and conjugated curcuminoid species in brain tissue has not been fully resolved. Therefore, improved systemic bioavailability should not automatically be interpreted as proof of effective delivery of pharmacologically relevant concentrations of free curcumin to the brain [63,102,110].

For instance, a 2024 study evaluated neuropathological outcomes and directly addressed the bioavailability issue using a bioavailable micellar curcumin formulation in an AD mouse model [100]. Similarly, nanoparticle-based curcumin formulations have demonstrated improved pharmacokinetic properties, enhanced brain accumulation, and favorable pathological and cognitive outcomes in AD-related animal models compared to conventional curcumin preparations, which supports the potential value of delivery-based strategies in overcoming pharmacokinetic limitations [122]. The interpretation of mechanistic studies is also influenced by this formulation-centered viewpoint.

Due to curcumin’s low bioavailability and quick metabolism, many in vitro studies employ concentrations that might not be possible in human plasma or brain tissue following traditional oral administration. Therefore, unless pharmacokinetic data shows achievable exposure in relevant tissues, biological effects seen in cell cultures should be regarded as mechanistic evidence. The same caution applies to animal studies: when the formulation, dose, route of administration, and tissue exposure are clearly reported, positive results are more translationally informative [63]. Therefore, current formulation research is crucial to figuring out whether curcumin’s multi-target mechanisms can become clinically meaningful, not just a technical supplement to curcumin biology. Future research should evaluate whether a formulation improves exposure to pharmacologically relevant curcumin species in the brain and results in repeatable biomarker changes in addition to whether it raises circulating curcuminoid levels [110]. Curcumin should be viewed as a promising but formulation-limited compound with significant preclinical support and unresolved clinical translational potential until such evidence becomes available.

In addition, variability among in vivo findings should be interpreted in the context of substantial methodological heterogeneity across studies. Differences in curcumin formulations, oral bioavailability, pharmacokinetic profiles, dosing regimens, treatment duration, and routes of administration may significantly influence biological outcomes. Furthermore, experimental models of Alzheimer’s disease differ in the pathological features they reproduce, including amyloid deposition, tau pathology, neuroinflammation, and cognitive impairment, which may further contribute to divergent results. Therefore, inconsistencies observed across in vivo studies do not necessarily indicate an absence of therapeutic activity but may instead reflect differences in drug exposure, experimental design, and model-specific responses. Greater standardization of formulations, pharmacokinetic assessment, and study design will be important for improving reproducibility and translational interpretation of future investigations [63,64,110].

A representative overview of experimental and clinical studies evaluating curcumin in Alzheimer’s disease and related dementia models is presented in Table 1.

### 4.11. Overall Assessment

When considered collectively, the literature indicates that curcumin is one of the plant-derived polyphenols that has been studied the most in relation to Alzheimer’s disease. Its biological activity encompasses a number of interrelated domains, such as mitochondrial function, cellular stress-response pathways, neuroinflammation modulation, oxidative stress regulation, interference with amyloid-β aggregation, and more speculative effects on tau pathology [81,88,89,90].

Preclinical and mechanistic research provides the best evidence base. Curcumin’s interactions with inflammatory mediators, redox-sensitive signaling pathways, and amyloidogenic proteins are thoroughly explained by in vitro research. In vivo studies, which more reliably show integrated biological effects, such as improvements in cognitive function along with decreases in oxidative stress and neuroinflammatory markers across various experimental models, support these findings [58,68,72]. Heterogeneity in model systems, experimental setup, and outcome measures, however, continues to be a significant constraint. Clinical evidence, on the other hand, is still sparse, inconsistent, and heavily influenced by formulation. Despite acceptable safety profiles, early randomized controlled trials in AD patients did not show clear therapeutic efficacy [105].

Curcumin may have quantifiable biological or cognitive effects in humans, according to more recent research employing bioavailability-enhanced formulations, but these results are still conflicting and insufficient to prove clinical efficacy. This disparity is indicative of a larger translational gap between strong preclinical activity and weak or equivocal results in human research. Crucially, pharmacokinetic limitations continue to be a major problem. The interpretation of both mechanistic and clinical findings is complicated by poor solubility, rapid metabolism, and limited systemic exposure. These factors emphasize the significance of formulation in determining biological relevance [134,135].

Overall, curcumin can be considered a well-characterized multi-target compound with promising preclinical evidence and strong mechanistic support; however, there is insufficient and formulation-dependent clinical data to support firm conclusions about its therapeutic efficacy in AD. Therefore, curcumin is best positioned as a promising lead compound whose relevance lies in its ability to modulate multiple interconnected pathogenic pathways rather than as a validated anti-AD therapy. Its main unresolved challenge is the translation of preclinical findings into robust and repeatable clinical benefits.

## 5. Bisdemethoxycurcumin as an Emerging Curcuminoid Candidate

Curcuma longa contains a naturally occurring curcuminoid called bisdemethoxycurcumin (also called BDMC in some formulation studies), which shares structural similarities with curcumin. BDMC is still far less studied than curcumin, which predominates in the literature on Alzheimer’s disease. This makes it especially intriguing in the context of this review: BDMC can be positioned as an emerging curcuminoid that might help advance the field beyond traditional curcumin-centered approaches rather than as a curcumin substitute.

### 5.1. Rationale for Considering BDMC in AD

There are two primary justifications for taking BDMC into account in AD. First, BDMC and curcumin have a number of biologically significant characteristics in common, such as documented neuroprotective, anti-inflammatory, and antioxidant effects [136,137]. Second, the research on AD that is currently available indicates that BDMC may activate processes that are particularly pertinent to the biology of AD today, such as oxidative stress regulation, SIRT1/AMPK signaling, autophagy, astrocyte senescence, amyloid-β clearance, and formulation-dependent brain delivery [138,139]. This is significant because the field of curcumin is moving away from straightforward antioxidant narratives and toward a more complex translational framework that includes glial biology, cellular resilience, proteostasis, and drug delivery. Because recent research has looked at BDMC in connection to autophagy-mediated reduction of senescence and amyloid pathology rather than just as a general antioxidant, it fits nicely into this more recent framework.

### 5.2. Structural Characteristics and Structure–Activity Considerations of Bisdemethoxycurcumin

Bisdemethoxycurcumin (BDMC) differs structurally from curcumin by the absence of both methoxy (-OCH_3_) substituents on the aromatic rings while retaining the phenolic hydroxyl groups and the central α,β-unsaturated β-diketone linker. Although this modification appears relatively simple, available evidence suggests that methoxy substitution significantly influences the physicochemical and biological properties of curcuminoids.

The removal of methoxy groups alters the electronic environment of the aromatic rings and may affect the stabilization of reactive intermediates formed during redox reactions. Comparative studies of curcuminoids indicate that methoxy substitution contributes to antioxidant behavior and influences oxidative transformation pathways [140,141]. Consequently, BDMC should not be regarded merely as a less substituted analog of curcumin but rather as a structurally distinct curcuminoid with potentially different redox characteristics and biological interactions.

One of the most consistently reported consequences of methoxy-group removal is enhanced chemical stability. Native curcumin undergoes relatively rapid degradation under physiological and alkaline conditions [142], whereas BDMC has been shown to be more resistant to autoxidation and spontaneous chemical transformation than curcumin [143]. This increased stability may prolong the persistence of the parent compound in biological systems and could partially contribute to its distinct pharmacological profile. However, greater chemical stability should not automatically be interpreted as improved therapeutic efficacy, as bioavailability, metabolism, and tissue distribution remain critical determinants of biological activity.

Structure–activity relationship studies further suggest that aromatic substitution patterns influence molecular target engagement. Experimental evidence indicates that curcuminoids differing in methoxy-group content may exhibit distinct effects on inflammatory signaling pathways, including NF-κB-associated responses [144]. Such observations support the notion that relatively small structural differences within the curcuminoid family may translate into measurable differences in biological activity.

In the context of Alzheimer’s disease, these structural considerations may be relevant to the reported effects of BDMC on oxidative stress regulation, AMPK/SIRT1 signaling, autophagy, astrocyte senescence, and amyloid-β clearance. Nevertheless, current evidence remains limited, and most available studies have not directly compared BDMC and curcumin under identical experimental conditions. Therefore, definitive structure–activity relationships cannot yet be established. At present, BDMC is best viewed as a structurally distinct curcuminoid whose modified aromatic substitution pattern may contribute to enhanced chemical stability and potentially different pathway engagement relative to curcumin.

### 5.3. Mechanistic Pathways, Cellular Targets, and Translational Considerations of BDMC

One important mechanistic study used human neuroblastoma cells exposed to Aβ1–42 to study BDMC in an in vitro AD-related model [136]. In this model, BDMC enhanced antioxidant capacity, including glutathione-related activity and superoxide dismutase, and increased cell survival. Additionally, the study found that silent information regulator 1 (SIRT1) expression and AMP-activated protein kinase (AMPK) phosphorylation were elevated and that BDMC’s protective effects were diminished when AMPK or SIRT1 were pharmacologically inhibited. These findings lend credence to the hypothesis that BDMC protects against Aβ-associated cellular stress through AMPK/SIRT1-related mechanisms rather than through direct antioxidant activity alone.

Additionally, BDMC has been tested in vivo in the double-transgenic mouse model for Alzheimer’s disease research (APP/PS) [138]. In that study, BDMC was given intracerebroventricularly to the mice, and then it was determined whether SIRT1 signaling was involved in the effects. The study found that while treatment with the SIRT1 inhibitor (EX527) attenuated these effects, BDMC reduced oxidative stress-related pathology and improved AD-related outcomes. This suggests that SIRT1 plays a part in the neuroprotective effects of BDMC in an animal model relevant to AD. Because the paper employs the AD mouse model, it offers more robust disease-context support than cell-based research. However, intracerebroventricular administration circumvents the typical pharmacokinetic barriers that restrict oral or systemic delivery, which is a significant translational limitation.

The relationship between autophagy, astrocyte senescence, and Aβ clearance represents a particularly intriguing emerging avenue in BDMC research. A recent study investigated whether BDMC could pharmacologically induce autophagy and attenuate senescence-associated pathology in primary astrocytes and in 3xTg-AD mice [139]. The authors reported that AD-related pathology was associated with increased cellular senescence and impaired autophagic activity, whereas BDMC treatment was accompanied by enhanced expression of several autophagy-related proteins, including phosphorylated AMPK (pAMPK), phosphorylated UNC-51-like kinase 1 (pULK1), FIP200, Beclin-1 (BECN1), ATG5, and ATG7, together with reduced mammalian target of rapamycin (mTOR) activity and decreased SQSTM1/p62 expression. BDMC treatment was also associated with reductions in multiple senescence-related markers, including p16INK4A, p38, p53, and senescence-associated β-galactosidase activity. Importantly, BDMC suppressed key components of the senescence-associated secretory phenotype (SASP), reducing IL-6 and TNF-α secretion in senescent astrocytes and decreasing IL-6, TNF-α, and IL-1β levels in the brains of 3xTg-AD mice. Mechanistically, the knockdown of AMPK markedly attenuated both autophagy induction and the anti-senescent effects of BDMC, which supports the involvement of AMPK-dependent signaling in these responses. Enhanced autophagic activity was accompanied by improved astrocytic clearance of exogenously administered Aβ and reduced hippocampal amyloid burden in vivo. These findings are particularly noteworthy because they shift attention beyond neuron-centered mechanisms and highlight astrocytes as active contributors to amyloid homeostasis, neuroinflammation, and cellular senescence in AD pathology. Furthermore, the study aligns with the growing concept that impaired autophagy and senescence-associated dysfunction may contribute to neurodegenerative progression and, therefore, represent potential therapeutic targets in AD [139].

The apparent contrast between the intracerebroventricular administration study and the peripheral administration study warrants careful interpretation. In the APP/PS mouse model, BDMC was delivered directly into the cerebral ventricles, thereby bypassing gastrointestinal absorption, systemic metabolism, and blood–brain barrier transport limitations [138]. Such an approach is valuable for mechanistic investigations because it allows direct assessment of target engagement within the central nervous system. In contrast, the 3xTg-AD study employed peripheral administration and reported neuropathological, molecular, and behavioral effects consistent with central activity, including reduced hippocampal amyloid burden and modulation of autophagy- and senescence-related pathways [139]. However, the two studies cannot be directly compared with respect to brain exposure because neither was designed as a pharmacokinetic investigation. Together, they suggest that BDMC possesses biological activity relevant to AD pathology, but they also highlight the continuing need for delivery strategies capable of achieving reproducible and therapeutically meaningful brain exposure following non-invasive administration. This issue remains particularly important given the limited aqueous solubility, low oral bioavailability, and rapid metabolism characteristic of curcuminoids.

Similar to curcumin, BDMC has significant formulation and delivery issues. BDMC is nearly insoluble in water, poorly absorbed, and quickly degraded, according to a study on BDMC-loaded H-ferritin nanocages [137]. In order to improve solubility, stability, and delivery, the authors created an H-ferritin nanocage formulation. Peripheral blood mononuclear cells from AD patients showed transcriptomic alterations as a result of the formulation crossing an in vitro BBB model; pathway analysis revealed effects on chemokines and macrophage activation. While stressing that in vivo confirmation is still necessary, the authors concluded that BDMC-HFn enhanced drug stability and modulated inflammation-related gene expression. That study provides preliminary evidence supporting the potential importance of formulation in determining the translational relevance of curcuminoids, suggesting that delivery may play a more integral role than previously considered. In addition, it highlights a possible connection between delivery strategies, AD-related inflammatory pathways, and curcuminoid chemistry. However, these findings should be interpreted with caution, as the study relied on an in vitro BBB model and PBMC transcriptomic analysis rather than clinical outcomes and, therefore, represents early translational evidence rather than confirmation of therapeutic efficacy.

### 5.4. Translational Positioning of BDMC in Alzheimer’s Disease

Instead of being positioned as a proven therapeutic agent for AD, BDMC should be considered an emerging curcuminoid candidate. The available literature suggests several potentially relevant mechanisms, including protection against Aβ-induced cellular stress, modulation of oxidative stress via AMPK/SIRT1-related pathways, reduction of senescence-associated astrocyte dysfunction, stimulation of autophagy, facilitation of Aβ clearance, and formulation-dependent anti-inflammatory effects.

These mechanisms align with the overarching theme of this review, particularly the concept that plant-derived compounds may act through interconnected glial, redox, neuroinflammatory, and proteostatic pathways. In addition, formulation-focused studies suggest that delivery strategies may influence the translational potential of BDMC, potentially linking curcuminoid chemistry with AD-related inflammatory pathways. However, such findings should be interpreted with caution, as they are primarily based on in vitro BBB models and transcriptomic analyses rather than clinical outcomes and, therefore, represent early translational observations rather than definitive evidence of therapeutic efficacy.

Despite these promising mechanistic insights, the overall body of evidence remains limited. Compared to curcumin, BDMC has been investigated far less extensively, lacks clinical validation in AD, and much of the available data is restricted to cell-based systems, animal models, or formulation-focused studies. Its primary value in the present manuscript is, therefore, conceptual and strategic: BDMC illustrates how exploration of structurally related curcuminoids with distinct mechanistic profiles and potentially improved formulation characteristics may help advance the field beyond canonical curcumin.

Overall, BDMC represents a promising but still early-stage candidate in AD research. Its emerging profile—linking curcuminoid biology to autophagy, astrocyte senescence, Aβ clearance, oxidative stress regulation, and formulation-dependent brain delivery—highlights its potential relevance. Nevertheless, it should currently be regarded as a mechanistically supported preclinical compound rather than a clinically validated therapeutic option.

**Figure 1:**
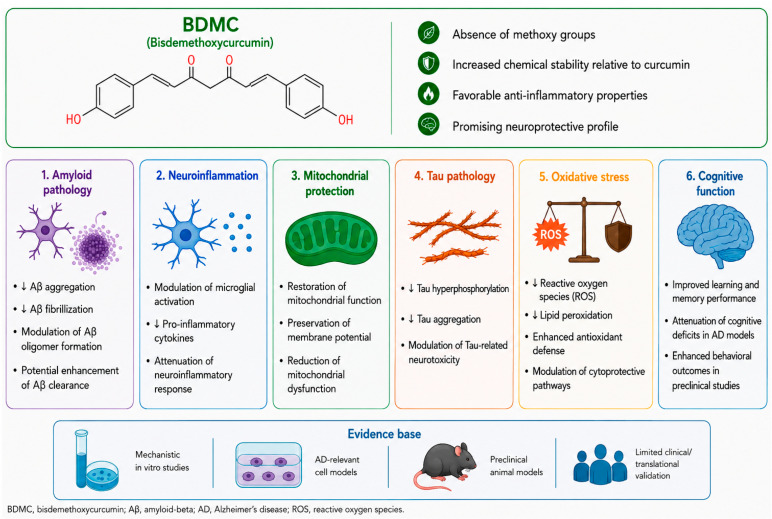
Mechanistic overview of the potential neuroprotective effects of bisdemethoxycurcumin in Alzheimer’s disease.

The multifaceted mechanisms potentially responsible for the neuroprotective activity of BDMC are summarized in Figure 1.

## 6. Conclusions and Future Perspectives

Alzheimer’s disease is a complex, multifactorial neurodegenerative disorder driven by interconnected processes, including neuroinflammation, oxidative stress, protein aggregation, mitochondrial dysfunction, and impaired cellular homeostasis. This complexity has shifted therapeutic strategies away from single-target approaches toward compounds capable of modulating multiple pathogenic pathways simultaneously.

In this context, curcumin remains one of the most extensively studied plant-derived polyphenols in AD research. The available evidence consistently demonstrates a broad spectrum of biological activity, including modulation of neuroinflammatory signaling, regulation of oxidative stress, interference with amyloid-β aggregation, and, to a lesser extent, effects on tau-related processes. These findings are strongly supported by mechanistic and preclinical studies, positioning curcumin as a prototypical multi-target compound within the AD research landscape.

Despite this strong experimental foundation, clinical translation remains limited. Human studies have yielded modest or inconclusive results, and therapeutic efficacy in AD has not been definitively established. A major contributor to this discrepancy is poor bioavailability, characterized by low aqueous solubility, rapid metabolism, and limited systemic and brain exposure. Consequently, curcumin exemplifies a compound with substantial mechanistic promise but unresolved translational challenges.

The increasing emphasis on formulation science has further refined the interpretation of curcumin’s therapeutic potential. Advances in delivery systems, including nanoparticles, micelles, and phospholipid complexes, have demonstrated improved pharmacokinetic profiles in preclinical and early clinical studies. However, it remains unclear whether these improvements translate into clinically meaningful outcomes. Future research should, therefore, focus on establishing whether enhanced formulations achieve therapeutically relevant brain concentrations and produce reproducible effects on validated disease biomarkers.

Within this evolving framework, structurally related curcuminoids, such as bisdemethoxycurcumin, have emerged as potential next-generation candidates. Preliminary evidence suggests that BDMC may modulate oxidative stress, Aβ-related toxicity, autophagy, and astrocyte senescence, while also benefiting from emerging formulation strategies. However, the current evidence base remains largely preclinical, and its clinical relevance has yet to be established.

Taken together, the available literature supports a reframing of curcumin not as a clinically validated therapeutic agent but rather as a mechanistically rich benchmark compound that has contributed to defining the multi-target paradigm in AD research. Its primary value lies in illustrating how plant-derived molecules can interact with interconnected pathological networks, while its limitations highlight key barriers to translation, including pharmacokinetics, formulation, and study design. From a therapeutic perspective, the available evidence supports the growing view that Alzheimer’s disease is unlikely to respond optimally to single-target interventions. Although current clinical data do not justify the routine use of curcumin in AD management, its ability to simultaneously modulate oxidative stress, neuroinflammation, protein aggregation, mitochondrial dysfunction, and cellular senescence provides further support for multi-target therapeutic strategies in AD. Consequently, curcumin may be most appropriately viewed as a potential adjunctive rather than a stand-alone intervention, particularly within future combination-based approaches designed to address the multifactorial nature of AD [5]. Furthermore, the available evidence suggests that therapeutic benefits may be more likely when interventions are introduced during earlier stages of disease progression, before extensive neuropathological damage has become established [85].

Accordingly, future research priorities should be reframed as a translational roadmap rather than as general methodological recommendations. First, future clinical studies should use standardized and analytically characterized formulations. At minimum, this should include clear reporting of the curcuminoid composition, delivery technology, dose, administration schedule, metabolic profile, and pharmacokinetic parameters. Particular attention should be paid to the distinction between total curcuminoids, conjugated metabolites, and pharmacologically relevant unconjugated curcumin, because recent pharmacokinetic analyses indicate that enhanced systemic exposure does not necessarily translate into meaningful circulating levels of unconjugated curcumin [63,109]. Therefore, future trials should not only compare formulations by nominal dose or total curcuminoid exposure but should also define reproducible exposure–response relationships and, if possible, establish formulation-specific plasma thresholds for unconjugated curcumin and relevant metabolites.

Second, future trials should incorporate biomarker-based confirmation of target engagement rather than relying primarily on cognitive endpoints. Blood-based biomarkers are increasingly recognized as scalable tools for detecting and monitoring AD-related pathology, particularly when integrated with established CSF or PET-based measures [145]. Among the currently available candidates, plasma phosphorylated tau 217 (p-tau217) should be prioritized as a marker of AD-type tau pathology and disease stratification, given its strong diagnostic performance across AD and non-AD neurodegenerative disorders [146]. Plasma Aβ42/Aβ40 may be useful for assessing amyloid-related biological context and participant selection, particularly when measured using high-precision or mass spectrometry-based approaches [147,148]. Neurofilament light chain (NfL) should be considered as a marker of neuroaxonal injury and disease progression, although it is not specific to AD pathology [149]. In addition, plasma glial fibrillary acidic protein (GFAP) may provide information on the astroglial activation and neuroinflammatory components of AD biology [150].

Third, because curcumin and BDMC are primarily discussed as modulators of redox balance, neuroinflammation, glial responses, and proteostasis, future multicenter studies should include mechanistic biomarker panels rather than single readouts. In addition to p-tau217, Aβ42/Aβ40, NfL, and GFAP, exploratory endpoints may include inflammatory cytokines, oxidative stress markers, and extracellular vesicle-based biomarkers reflecting CNS or glial cell-derived signaling. CNS-derived extracellular vesicles are increasingly investigated as potential biomarker sources in neurodegenerative diseases, but their use still requires methodological standardization, particularly with respect to vesicle isolation, cellular origin assignment, and cross-study reproducibility [151]. Accordingly, microglial- or astrocyte-enriched extracellular vesicle markers should currently be regarded as exploratory tools rather than validated clinical endpoints.

Ultimately, definitive assessment of the therapeutic potential of curcumin and BDMC in Alzheimer’s disease will require adequately powered multicenter trials that combine optimized formulations, pharmacokinetic monitoring, biomarker-confirmed target engagement, and long-term clinical follow-up.

Overall, the findings reviewed in this manuscript contribute to a more integrated understanding of Alzheimer’s disease by highlighting the interconnected roles of oxidative stress, neuroinflammation, amyloid-β pathology, tau dysfunction, and mitochondrial impairment. The available evidence suggests that curcumin may serve as a useful model compound for investigating multi-target therapeutic strategies in AD, particularly given its ability to influence several disease-associated pathways simultaneously. Although the current clinical evidence remains insufficient to support routine therapeutic use, insights gained from curcumin research may help guide the development of next-generation multi-target interventions, optimized delivery systems, and biomarker-driven clinical trial designs [3,63,104,145].

Major challenges in clinical translation include poor and variable bioavailability, extensive metabolism, formulation-dependent pharmacokinetics, limited standardization across studies, and the scarcity of large, adequately powered clinical trials. In addition, heterogeneity in patient populations, treatment duration, dosing regimens, and outcome measures complicates direct comparisons between studies and may contribute to inconsistent clinical findings. Addressing these limitations will require harmonized formulations, rigorous pharmacokinetic characterization, and biomarker-guided clinical trial designs capable of confirming target engagement and biological efficacy [63,64,110,145].

In summary, curcumin represents both the promise and the limitations of plant-derived multi-target compounds in AD. While it has a significantly advanced mechanistic understanding, its clinical potential remains constrained. Bridging this gap will require a shift from descriptive mechanistic studies toward integrative, translational research. In this context, emerging curcuminoid strategies, including BDMC, represent a promising but still exploratory direction for future investigation.

## Figures and Tables

**Table 1:** Representative studies on curcumin in Alzheimer’s disease and related dementia models.

Reference	Type of Evidence	Main Finding(s)	Main Mechanism(s) Domain
[78]	In vivo, APPSw Tg+ and Tg− mice	Reduced oxidative damage, IL-1β and amyloid pathology	Anti-inflammatory; antioxidant; anti-amyloid
[123]	In vivo, Aβ-infused rat	Attenuated Aβ-induced oxidative/inflammatory damage	Anti-inflammatory; antioxidant
[124]	In vitro, PC12 rat pheochromocytoma cells	Protected neuronal cells against Aβ-induced toxicity	Neuroprotection; antioxidant
[76]	In vitro, β-amyloid fibrils	Inhibited Aβ aggregation and destabilized fibrils	Anti-amyloid aggregation
[79]	In vitro model of Aβ fibrilization + in vivo Tg2576 mice	Inhibited Aβ oligomers/fibrils, bound plaques, reduced amyloid burden	Anti-amyloid; plaque binding
[125]	Ex vivo, human AD macrophages	Curcuminoids enhanced Aβ uptake by macrophages	Immunomodulation; Aβ clearance
[126]	In vivo imaging, APPswe/PS1dE9 mice	Curcumin labeled amyloid deposits in vivo	Amyloid binding/imaging
[101]	In vivo Tg2576 APP *sw* mice	Curcumin reduced neuroinflammation and AD-like pathology	Anti-inflammatory; anti-amyloid
[127]	Clinical pilot randomized controlled trial (RCT), AD patients	Generally safe but no clear clinical efficacy signal	Clinical tolerability; bioavailability limitation
[128]	Ex vivo, human AD macrophages	Curcuminoids + vitamin D enhanced Aβ phagocytosis in selected patients	Innate immunity; Aβ clearance
[129]	In vivo, amyloid-β peptide-infused rats	Curcuminoids improved memory and synaptic protein expression	Synaptic plasticity; cognition
[130]	Ex vivo, human AD macrophages	β-1,4-mannosyl-glycoprotein 4-β-N-acetylglucosaminyltransferase (MGAT3) response may distinguish curcuminoid-responsive subgroups	Biomarker; immunomodulation
[105]	Clinical RCT, AD patients	Oral curcumin was tolerated but showed limited efficacy, likely due to low bioavailability	Clinical translation; bioavailability
[131]	In vivo, Aβ1–42 Sprague Dawley rats	Chronic curcumin improved spatial memory dose-dependently	Cognition; BDNF/synaptic pathways
[73]	In vivo, p25 mouse model	Reduced neuroinflammation, neurodegeneration and memory decline	Neuroinflammation; neurodegeneration; synaptic dysfunction; cognition
[84]	Clinical RCT, non-demented adults	Theracurmin improved memory/attention and reduced PET amyloid/tau signals	Amyloid/tau; cognition
[132]	Cell-based AD patient studies	Curcumin analogues modulated AD-related inflammatory and amyloidogenic pathways	Anti-inflammatory; amyloidogenesis
[72]	In vivo, Aβ1–42 hippocampal injection mouse model	Curcumin improved cognition and reduced inflammation, oxidative stress, neuronal damage and Aβ deposition	Neuroinflammation; oxidative stress; AMPK signaling; anti-apoptotic effects
[100]	In vivo, APPswe/PS1dE9 mice on a C57BL/6J background	A bioavailable curcumin formulation reduced some AD-related pathological features but was associated with increased neuroinflammatory responses	Neuroinflammation; amyloid pathology; bioavailability-related effects
[99]	In vitro biophysical study	Curcumin attenuated Aβ oligomer binding to anionic lipid membranes, potentially reducing membrane-associated toxicity	Anti-amyloid effects; membrane interaction modulation
[90]	In vitro, tau aggregation/phosphorylation model	The co-amorphous curcumin–artemisinin formulation attenuated Tau aggregation and hyperphosphorylation	Tau aggregation; tau phosphorylation; neuroprotection
[133]	In vivo, 3xTg-AD model	Curcumin improved AD-related pathological and cognitive alterations in a metabolically stressed AD model	Metabolic stress; neuroinflammation; amyloid/tau

## Data Availability

No new data were created or analyzed in this study. Data sharing is not applicable to this article.
